# 1-(3,4-Dichloro­benz­yl)-3-methyl­quinolin-1-ium 7,7,8,8-tetra­cyano­quinodimethanide

**DOI:** 10.1107/S1600536810002862

**Published:** 2010-01-30

**Authors:** Guang-Xiang Liu, Chun-You Zhang

**Affiliations:** a Anhui Key Laboratory of Functional Coordination Compounds, School of Chemistry and Chemical Engineering, Anqing Normal University, Anqing 246003, People’s Republic of China

## Abstract

In the title salt, C_17_H_14_Cl_2_N^+^·C_12_H_4_N_4_^−^, cations and anions stack along the *a* axis into segregated columns by π–π stacking inter­actions, with alternating centroid–centroid separations of 3.5957 (7) and 3.7525 (7) Å for the cation column and 3.4252 (6) and 4.1578 (7) Å for the anion column. In the cation, the dihedral angle between the benzene ring and the quinoline ring system is 76.35 (4)°. The crystal packing is stabilized by inter­columnar C—H⋯N hydrogen bonds.

## Related literature

For general background to the planar organic mol­ecule 7,7,8,8-tetra­cyano­quinodimethane, see: Alonso *et al.* (2005 ▶); Madalan *et al.* (2002 ▶); Liu *et al.* (2008 ▶). For the role played by the size and shape of the counter-cations in determining the ground-state properties of the resulting materials, see: Ren, Meng *et al.* (2002 ▶); Ren *et al.* (2003 ▶); Ren, Chen *et al.* (2002 ▶). For related structures, see: Liu *et al.* (2005 ▶).
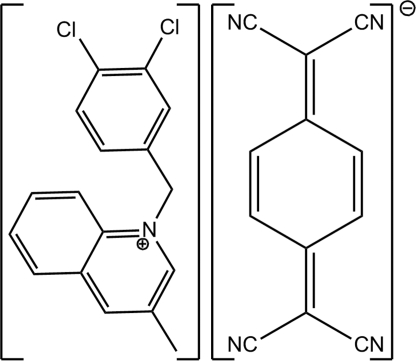


## Experimental

### 

#### Crystal data

C_17_H_14_Cl_2_N^+^·C_12_H_4_N_4_^−^
*M* *_r_* = 507.38
Monoclinic, 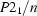

*a* = 7.0795 (14) Å
*b* = 18.704 (4) Å
*c* = 18.608 (4) Å
β = 95.286 (2)°
*V* = 2453.4 (9) Å^3^
*Z* = 4
Mo *K*α radiation
μ = 0.29 mm^−1^
*T* = 293 K
0.26 × 0.16 × 0.12 mm

#### Data collection

Bruker SMART APEX CCD area-detector diffractometer
Absorption correction: multi-scan (*SADABS*; Bruker, 2000 ▶) *T*_min_ = 0.928, *T*_max_ = 0.966
18184 measured reflections
4580 independent reflections
3680 reflections with *I* > 2σ(*I*)
*R*_int_ = 0.027

#### Refinement

*R*[*F* ^2^ > 2σ(*F* ^2^)] = 0.039
*wR*(*F* ^2^) = 0.101
*S* = 1.03
4580 reflections
326 parameters
H-atom parameters constrained
Δρ_max_ = 0.25 e Å^−3^
Δρ_min_ = −0.29 e Å^−3^

### 

Data collection: *SMART* (Bruker, 2000 ▶); cell refinement: *SAINT* (Bruker, 2000 ▶); data reduction: *SAINT*; program(s) used to solve structure: *SHELXS97* (Sheldrick, 2008 ▶); program(s) used to refine structure: *SHELXL97* (Sheldrick, 2008 ▶); molecular graphics: *SHELXTL* (Sheldrick, 2008 ▶); software used to prepare material for publication: *SHELXTL*.

## Supplementary Material

Crystal structure: contains datablocks I, global. DOI: 10.1107/S1600536810002862/rz2411sup1.cif

Structure factors: contains datablocks I. DOI: 10.1107/S1600536810002862/rz2411Isup2.hkl

Additional supplementary materials:  crystallographic information; 3D view; checkCIF report

## Figures and Tables

**Table 1:** Hydrogen-bond geometry (Å, °)

*D*—H⋯*A*	*D*—H	H⋯*A*	*D*⋯*A*	*D*—H⋯*A*
C20—H20⋯N3^i^	0.93	2.53	3.387 (3)	154
C19—H19*B*⋯N3^i^	0.97	2.51	3.432 (2)	158
C14—H14⋯N3^ii^	0.93	2.50	3.390 (3)	161
C15—H15⋯N1^iii^	0.93	2.45	3.348 (2)	163

Symmetry codes: (i) 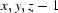; (ii) 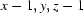; (iii) 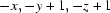.

**Fig. 1.**
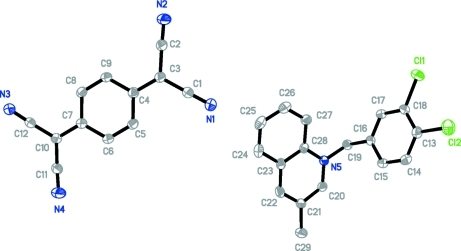
The asymmetric unit of the title compound, with the atom-numbering scheme. Displacement ellipsoids are drawn at the 30% probability level. Hydrogen atoms are omitted for clarity.

**Fig. 2.**
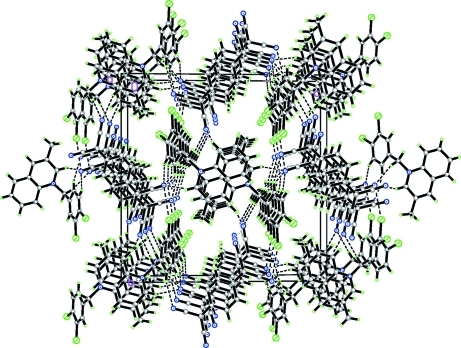
Packing diagram of the title compound viewed along the a axis. Hydrogen bonds are shown as dashed lines.

**Fig. 3.**
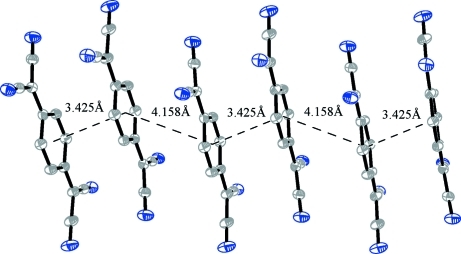
A side-view of the one-dimensional anion column in the title compound. Centroid-to-centroid distances (dashed lines) are in Å. Hydrogen atoms are omitted for clarity.

## supplementary crystallographic information

### Comment

The search for new compounds with promising electronic, and magnetic properties has prompted chemists to combine different spin carriers within the same molecular or supramolecular entity (Madalan *et al.*, 2002). One of the most extensively used radicals in these studies has been the planar organic molecule 7,7,8,8-tetracyanoquinodimethane, [C_8_H_4_(CN)_4_], TCNQ, since it shows a low reduction potential which makes it a suitable acceptor in charge-transfer processes. Another significant feature of this acceptor is its tendency to overlap its π-delocalized system with those of neighbouring molecules to form stacks with different degrees of electron delocalization (Alonso *et al.*, 2005). Previous work has shown that molecular stacks of charge-transfer salts exhibit low-dimensional properties in some cases, which have intriguing anisotropic magnetic, electronic and structural characteristics (Ren, Meng *et al.*, 2002; Ren *et al.*, 2003; Liu *et al.*, 2005). Furthermore, the size and shape of the counter-cations play an important role in determining the ground-state properties of the resulting materials (Ren, Chen *et al.*, 2002; Liu *et al.*, 2008). As a result, charge-transfer salts consisting of the TCNQ anion and benzylpyridinium cations could offer the possibility of systematically studying the fundamental relationship between the stack structure and the size and steric properties of substituent groups. In this communication, we report the crystal structure of the title complex.

The asymmetric unit of the title compound contains one (C_17_H_14_Cl_2_N)^+^ cation and one [C_8_H_4_(CN)_4_]^-^ anion (Fig. 1). Anions and cations stack into completely segregated columns along the *a* axis, as illustrated in Fig. 2. Within an anion column, the strongly bound unit [(TCNQ)_2_]^2-^ is formed by π–π stacking interactions with a centroid-to-centroid distance of 3.4252 (6) Å, and adjacent units are displaced relative to each other along the direction of the shorter molecular axis of TCNQ with centroid-to-centroid separations of 4.1578 (7) Å (Fig. 3). Stacking within the cation column is also governed by π–π stacking interactions with alternating centroid-to-centroid distances 3.5957 (7) and 3.7525 (7) Å. The (C_17_H_14_Cl_2_N)^+^ cation assumes a Λ-shaped conformation, with a dihedral angle between the benzene ring and the quinoline ring system of 76.35 (4)°. The crystal packing is stabilized by C—H···N intercolumar linkages (Table 1).

### Experimental

1-(3,4-Dichlorobenzyl)-3-methylquinolin-1-ium iodide was prepared by the direct combination of 1:1 molar equivalents of 1-(3,4-dichlorobenzyl)-3-methylquinolin-1-ium chloride and NaI in a warm solution in acetone at 313 K. A white precipitate was formed (NaCl), which was filtered off, and a white microcrystalline product was obtained by evaporating the filtrate. 1:1 Molar equivalents of 1-(3,4-dichlorobenzyl)-3-methylquinolin-1-ium iodide and 7,7,8,8-tetracyanoquinodimethane (TCNQ) were mixed directly in methanol, and the mixture was refluxed for 12 h. The black microcrystalline product which formed was filtered off, washed with MeOH and dried *in vacuo*. Single crystals of the title compound suitable for X-ray structure analysis were obtained by diffusing diethyl ether into a MeCN solution.

### Refinement

H atoms were positioned geometrically, with C—H = 0.93, 0.97 and 0.96 Å for aromatic, methylene and methyl H atoms, respectively, and constrained to ride on their parent atoms, with *U*_iso_(H) = 1.2 *U*_eq_(C) or 1.5 *U*_eq_(C) for methyl H atoms.

### Crystal data

**Table:**

C_17_H_14_Cl_2_N^+^·C_12_H_4_N_4_^−^	*F*(000) = 1044
*M**_r_* = 507.38	*D*_x_ = 1.374 Mg m^−^^3^
Monoclinic, *P*2_1_/*n*	Mo *K*α radiation, λ = 0.71073 Å
Hall symbol: -P 2yn	Cell parameters from 7732 reflections
*a* = 7.0795 (14) Å	θ = 2.4–27.6°
*b* = 18.704 (4) Å	µ = 0.29 mm^−^^1^
*c* = 18.608 (4) Å	*T* = 293 K
β = 95.286 (2)°	Block, black
*V* = 2453.4 (9) Å^3^	0.26 × 0.16 × 0.12 mm
*Z* = 4	

### Data collection

**Table:**

Bruker SMART APEX CCD area-detector diffractometer	4580 independent reflections
Radiation source: sealed tube	3680 reflections with *I* > 2σ(*I*)
graphite	*R*_int_ = 0.027
phi and ω scans	θ_max_ = 25.5°, θ_min_ = 1.6°
Absorption correction: multi-scan (*SADABS*; Bruker, 2000)	*h* = −8→8
*T*_min_ = 0.928, *T*_max_ = 0.966	*k* = −22→22
18184 measured reflections	*l* = −22→22

### Refinement

**Table:**

Refinement on *F*^2^	Primary atom site location: structure-invariant direct methods
Least-squares matrix: full	Secondary atom site location: difference Fourier map
*R*[*F*^2^ > 2σ(*F*^2^)] = 0.039	Hydrogen site location: inferred from neighbouring sites
*wR*(*F*^2^) = 0.101	H-atom parameters constrained
*S* = 1.03	*w* = 1/[σ^2^(*F*_o_^2^) + (0.0415*P*)^2^ + 0.9033*P*] where *P* = (*F*_o_^2^ + 2*F*_c_^2^)/3
4580 reflections	(Δ/σ)_max_ = 0.001
326 parameters	Δρ_max_ = 0.25 e Å^−^^3^
0 restraints	Δρ_min_ = −0.29 e Å^−^^3^

### Special details

**Table:**

Geometry. All e.s.d.'s (except the e.s.d. in the dihedral angle between two l.s. planes) are estimated using the full covariance matrix. The cell e.s.d.'s are taken into account individually in the estimation of e.s.d.'s in distances, angles and torsion angles; correlations between e.s.d.'s in cell parameters are only used when they are defined by crystal symmetry. An approximate (isotropic) treatment of cell e.s.d.'s is used for estimating e.s.d.'s involving l.s. planes.
Refinement. Refinement of *F*^2^ against ALL reflections. The weighted *R*-factor *wR* and goodness of fit *S* are based on *F*^2^, conventional *R*-factors *R* are based on *F*, with *F* set to zero for negative *F*^2^. The threshold expression of *F*^2^ > σ(*F*^2^) is used only for calculating *R*-factors(gt) *etc*. and is not relevant to the choice of reflections for refinement. *R*-factors based on *F*^2^ are statistically about twice as large as those based on *F*, and *R*- factors based on ALL data will be even larger.

### Fractional atomic coordinates and isotropic or equivalent isotropic displacement parameters (Å^2^)

**Table:**

	*x*	*y*	*z*	*U*_iso_*/*U*_eq_	
N5	0.29919 (19)	0.47148 (7)	0.39305 (7)	0.0366 (3)	
N1	−0.0533 (2)	0.39215 (9)	0.75827 (9)	0.0550 (4)	
N3	0.4950 (3)	0.53355 (8)	1.21413 (9)	0.0536 (4)	
N4	0.3739 (3)	0.70369 (9)	1.04907 (11)	0.0700 (5)	
N2	0.0918 (3)	0.22596 (9)	0.91236 (10)	0.0730 (6)	
Cl1	−0.08219 (9)	0.19603 (3)	0.25897 (3)	0.06871 (18)	
Cl2	−0.40722 (9)	0.30060 (3)	0.19589 (4)	0.0817 (2)	
C4	0.1467 (2)	0.41286 (9)	0.93789 (9)	0.0392 (4)	
C5	0.1399 (2)	0.48648 (9)	0.92029 (9)	0.0402 (4)	
H5	0.0852	0.5004	0.8751	0.048*	
C7	0.2918 (2)	0.51917 (9)	1.03746 (9)	0.0368 (4)	
C28	0.2600 (2)	0.45003 (9)	0.46162 (9)	0.0366 (4)	
C17	0.1152 (3)	0.31440 (9)	0.29677 (9)	0.0433 (4)	
H17	0.2095	0.2834	0.3158	0.052*	
C12	0.4384 (3)	0.55209 (9)	1.15743 (10)	0.0407 (4)	
C20	0.3127 (2)	0.54043 (9)	0.37601 (9)	0.0398 (4)	
H20	0.3386	0.5524	0.3294	0.048*	
C23	0.2461 (2)	0.50382 (10)	0.51399 (9)	0.0405 (4)	
C8	0.2976 (2)	0.44532 (9)	1.05546 (9)	0.0408 (4)	
H8	0.3498	0.4315	1.1010	0.049*	
C21	0.2898 (2)	0.59540 (9)	0.42521 (10)	0.0432 (4)	
C1	0.0043 (3)	0.37834 (9)	0.81630 (10)	0.0427 (4)	
C10	0.3650 (2)	0.57206 (9)	1.08701 (9)	0.0386 (4)	
C6	0.2107 (2)	0.53737 (9)	0.96747 (9)	0.0386 (4)	
H6	0.2059	0.5851	0.9535	0.046*	
C16	0.1436 (2)	0.38756 (9)	0.30184 (9)	0.0378 (4)	
C27	0.2366 (2)	0.37768 (9)	0.47945 (10)	0.0435 (4)	
H27	0.2430	0.3423	0.4446	0.052*	
C19	0.3282 (2)	0.41725 (9)	0.33621 (9)	0.0407 (4)	
H19A	0.4048	0.3784	0.3575	0.049*	
H19B	0.3972	0.4391	0.2992	0.049*	
C18	−0.0524 (3)	0.28720 (9)	0.26362 (10)	0.0451 (4)	
C14	−0.1659 (3)	0.40587 (10)	0.24047 (10)	0.0497 (5)	
H14	−0.2606	0.4368	0.2216	0.060*	
C22	0.2612 (2)	0.57597 (10)	0.49425 (10)	0.0453 (4)	
H22	0.2514	0.6113	0.5289	0.054*	
C11	0.3706 (3)	0.64519 (10)	1.06735 (10)	0.0454 (4)	
C9	0.2293 (3)	0.39456 (9)	1.00816 (9)	0.0430 (4)	
H9	0.2365	0.3467	1.0218	0.052*	
C13	−0.1939 (3)	0.33322 (10)	0.23607 (10)	0.0480 (5)	
C2	0.0867 (3)	0.28632 (10)	0.90231 (10)	0.0506 (5)	
C3	0.0775 (3)	0.36059 (9)	0.88739 (9)	0.0434 (4)	
C24	0.2156 (3)	0.48249 (12)	0.58499 (10)	0.0517 (5)	
H24	0.2092	0.5169	0.6207	0.062*	
C15	0.0019 (3)	0.43294 (9)	0.27275 (9)	0.0450 (4)	
H15	0.0202	0.4821	0.2751	0.054*	
C25	0.1956 (3)	0.41215 (12)	0.60159 (10)	0.0554 (5)	
H25	0.1759	0.3988	0.6485	0.066*	
C26	0.2045 (3)	0.35999 (11)	0.54832 (10)	0.0523 (5)	
H26	0.1882	0.3122	0.5601	0.063*	
C29	0.2965 (3)	0.67200 (10)	0.40124 (12)	0.0608 (6)	
H29A	0.2461	0.7022	0.4366	0.091*	
H29B	0.2222	0.6775	0.3558	0.091*	
H29C	0.4256	0.6853	0.3961	0.091*	

### Atomic displacement parameters (Å^2^)

**Table:**

	*U*^11^	*U*^22^	*U*^33^	*U*^12^	*U*^13^	*U*^23^
N5	0.0368 (8)	0.0358 (8)	0.0364 (7)	−0.0013 (6)	−0.0005 (6)	−0.0012 (6)
N1	0.0675 (11)	0.0504 (10)	0.0451 (10)	0.0036 (8)	−0.0050 (8)	−0.0007 (7)
N3	0.0688 (11)	0.0444 (9)	0.0465 (9)	0.0067 (8)	0.0004 (8)	0.0009 (7)
N4	0.0915 (15)	0.0396 (10)	0.0773 (13)	0.0033 (9)	−0.0012 (11)	0.0034 (9)
N2	0.1053 (16)	0.0408 (10)	0.0680 (12)	−0.0153 (10)	−0.0177 (11)	0.0083 (9)
Cl1	0.0850 (4)	0.0364 (3)	0.0817 (4)	−0.0103 (2)	−0.0086 (3)	−0.0031 (2)
Cl2	0.0627 (4)	0.0787 (4)	0.0976 (5)	−0.0133 (3)	−0.0252 (3)	−0.0025 (3)
C4	0.0411 (9)	0.0382 (9)	0.0382 (9)	−0.0040 (7)	0.0033 (7)	0.0021 (7)
C5	0.0450 (10)	0.0390 (9)	0.0363 (9)	0.0000 (8)	0.0018 (7)	0.0061 (7)
C7	0.0342 (9)	0.0363 (9)	0.0404 (9)	0.0004 (7)	0.0054 (7)	0.0012 (7)
C28	0.0310 (9)	0.0427 (9)	0.0353 (9)	−0.0002 (7)	−0.0012 (7)	0.0009 (7)
C17	0.0523 (11)	0.0343 (9)	0.0423 (10)	0.0044 (8)	−0.0010 (8)	0.0025 (7)
C12	0.0442 (10)	0.0334 (9)	0.0447 (10)	0.0002 (7)	0.0054 (8)	−0.0046 (8)
C20	0.0396 (9)	0.0380 (9)	0.0404 (9)	−0.0035 (7)	−0.0044 (7)	0.0026 (7)
C23	0.0328 (9)	0.0492 (10)	0.0384 (9)	−0.0004 (7)	−0.0030 (7)	−0.0046 (8)
C8	0.0460 (10)	0.0397 (9)	0.0359 (9)	−0.0007 (8)	−0.0010 (7)	0.0052 (7)
C21	0.0403 (10)	0.0379 (9)	0.0489 (10)	−0.0017 (7)	−0.0092 (8)	−0.0015 (8)
C1	0.0473 (10)	0.0361 (9)	0.0441 (11)	−0.0029 (8)	0.0015 (8)	−0.0026 (8)
C10	0.0391 (9)	0.0361 (9)	0.0407 (9)	0.0011 (7)	0.0034 (7)	−0.0001 (7)
C6	0.0416 (9)	0.0333 (9)	0.0410 (9)	0.0004 (7)	0.0046 (7)	0.0058 (7)
C16	0.0459 (10)	0.0363 (9)	0.0314 (8)	0.0009 (7)	0.0037 (7)	−0.0006 (7)
C27	0.0433 (10)	0.0419 (10)	0.0446 (10)	−0.0002 (8)	0.0008 (8)	0.0021 (8)
C19	0.0451 (10)	0.0389 (9)	0.0385 (9)	0.0009 (8)	0.0059 (8)	−0.0023 (7)
C18	0.0584 (11)	0.0331 (9)	0.0430 (10)	−0.0028 (8)	0.0006 (8)	−0.0006 (7)
C14	0.0530 (11)	0.0450 (11)	0.0497 (11)	0.0096 (9)	−0.0031 (9)	0.0058 (8)
C22	0.0395 (10)	0.0472 (11)	0.0476 (11)	0.0007 (8)	−0.0056 (8)	−0.0137 (8)
C11	0.0501 (11)	0.0392 (10)	0.0461 (10)	0.0029 (8)	−0.0004 (8)	−0.0043 (8)
C9	0.0545 (11)	0.0326 (9)	0.0411 (10)	−0.0022 (8)	0.0004 (8)	0.0063 (7)
C13	0.0489 (11)	0.0495 (11)	0.0440 (10)	−0.0041 (9)	−0.0034 (8)	−0.0010 (8)
C2	0.0652 (13)	0.0429 (11)	0.0415 (10)	−0.0122 (9)	−0.0072 (9)	0.0021 (8)
C3	0.0531 (11)	0.0378 (9)	0.0385 (9)	−0.0062 (8)	0.0004 (8)	0.0039 (7)
C24	0.0445 (11)	0.0706 (14)	0.0395 (10)	0.0008 (9)	0.0004 (8)	−0.0085 (9)
C15	0.0563 (11)	0.0333 (9)	0.0448 (10)	0.0022 (8)	0.0005 (8)	0.0018 (8)
C25	0.0482 (11)	0.0777 (15)	0.0402 (10)	0.0005 (10)	0.0042 (8)	0.0125 (10)
C26	0.0477 (11)	0.0560 (12)	0.0530 (12)	0.0001 (9)	0.0040 (9)	0.0145 (9)
C29	0.0718 (14)	0.0380 (10)	0.0697 (14)	−0.0017 (10)	−0.0090 (11)	−0.0013 (10)

### Geometric parameters (Å, °)

**Table:**

N5—C20	1.334 (2)	C8—H8	0.9300
N5—C28	1.390 (2)	C21—C22	1.368 (3)
N5—C19	1.493 (2)	C21—C29	1.502 (3)
N1—C1	1.148 (2)	C1—C3	1.415 (2)
N3—C12	1.147 (2)	C10—C11	1.417 (2)
N4—C11	1.147 (2)	C6—H6	0.9300
N2—C2	1.144 (2)	C16—C15	1.386 (2)
Cl1—C18	1.7195 (18)	C16—C19	1.507 (2)
Cl2—C13	1.7334 (19)	C27—C26	1.363 (3)
C4—C3	1.412 (2)	C27—H27	0.9300
C4—C5	1.415 (2)	C19—H19A	0.9700
C4—C9	1.424 (2)	C19—H19B	0.9700
C5—C6	1.359 (2)	C18—C13	1.383 (3)
C5—H5	0.9300	C14—C13	1.374 (3)
C7—C6	1.416 (2)	C14—C15	1.377 (3)
C7—C10	1.418 (2)	C14—H14	0.9300
C7—C8	1.421 (2)	C22—H22	0.9300
C28—C27	1.407 (2)	C9—H9	0.9300
C28—C23	1.410 (2)	C2—C3	1.417 (3)
C17—C16	1.385 (2)	C24—C25	1.362 (3)
C17—C18	1.382 (3)	C24—H24	0.9300
C17—H17	0.9300	C15—H15	0.9300
C12—C10	1.415 (2)	C25—C26	1.396 (3)
C20—C21	1.396 (2)	C25—H25	0.9300
C20—H20	0.9300	C26—H26	0.9300
C23—C22	1.405 (3)	C29—H29A	0.9600
C23—C24	1.416 (3)	C29—H29B	0.9600
C8—C9	1.353 (2)	C29—H29C	0.9600
			
C20—N5—C28	121.49 (14)	N5—C19—C16	112.36 (14)
C20—N5—C19	118.10 (14)	N5—C19—H19A	109.1
C28—N5—C19	120.42 (14)	C16—C19—H19A	109.1
C3—C4—C5	121.14 (15)	N5—C19—H19B	109.1
C3—C4—C9	122.19 (15)	C16—C19—H19B	109.1
C5—C4—C9	116.67 (15)	H19A—C19—H19B	107.9
C6—C5—C4	121.95 (16)	C13—C18—C17	119.92 (17)
C6—C5—H5	119.0	C13—C18—Cl1	121.14 (15)
C4—C5—H5	119.0	C17—C18—Cl1	118.93 (14)
C6—C7—C10	121.57 (15)	C13—C14—C15	120.20 (17)
C6—C7—C8	116.82 (15)	C13—C14—H14	119.9
C10—C7—C8	121.61 (15)	C15—C14—H14	119.9
N5—C28—C27	122.15 (15)	C21—C22—C23	121.46 (16)
N5—C28—C23	117.45 (15)	C21—C22—H22	119.3
C27—C28—C23	120.40 (16)	C23—C22—H22	119.3
C16—C17—C18	120.48 (16)	N4—C11—C10	177.7 (2)
C16—C17—H17	119.8	C8—C9—C4	121.36 (16)
C18—C17—H17	119.8	C8—C9—H9	119.3
N3—C12—C10	177.57 (19)	C4—C9—H9	119.3
N5—C20—C21	122.75 (17)	C14—C13—C18	119.85 (17)
N5—C20—H20	118.6	C14—C13—Cl2	119.25 (15)
C21—C20—H20	118.6	C18—C13—Cl2	120.89 (15)
C22—C23—C28	119.52 (16)	N2—C2—C3	178.0 (2)
C22—C23—C24	122.44 (17)	C4—C3—C1	122.31 (16)
C28—C23—C24	118.04 (17)	C4—C3—C2	122.77 (16)
C9—C8—C7	121.86 (16)	C1—C3—C2	114.80 (15)
C9—C8—H8	119.1	C25—C24—C23	120.81 (18)
C7—C8—H8	119.1	C25—C24—H24	119.6
C22—C21—C20	117.16 (16)	C23—C24—H24	119.6
C22—C21—C29	122.93 (17)	C14—C15—C16	120.64 (17)
C20—C21—C29	119.91 (17)	C14—C15—H15	119.7
N1—C1—C3	179.1 (2)	C16—C15—H15	119.7
C12—C10—C7	119.95 (15)	C24—C25—C26	120.09 (18)
C12—C10—C11	118.51 (15)	C24—C25—H25	120.0
C7—C10—C11	121.52 (15)	C26—C25—H25	120.0
C5—C6—C7	121.33 (15)	C27—C26—C25	121.35 (19)
C5—C6—H6	119.3	C27—C26—H26	119.3
C7—C6—H6	119.3	C25—C26—H26	119.3
C17—C16—C15	118.89 (16)	C21—C29—H29A	109.5
C17—C16—C19	120.51 (15)	C21—C29—H29B	109.5
C15—C16—C19	120.56 (15)	H29A—C29—H29B	109.5
C26—C27—C28	119.26 (18)	C21—C29—H29C	109.5
C26—C27—H27	120.4	H29A—C29—H29C	109.5
C28—C27—H27	120.4	H29B—C29—H29C	109.5
			
C3—C4—C5—C6	−178.09 (17)	C17—C16—C19—N5	−128.65 (17)
C9—C4—C5—C6	0.9 (3)	C15—C16—C19—N5	53.4 (2)
C20—N5—C28—C27	177.01 (15)	C16—C17—C18—C13	−0.8 (3)
C19—N5—C28—C27	−3.2 (2)	C16—C17—C18—Cl1	−179.64 (14)
C20—N5—C28—C23	−3.7 (2)	C20—C21—C22—C23	−3.1 (3)
C19—N5—C28—C23	176.08 (14)	C29—C21—C22—C23	176.76 (17)
C28—N5—C20—C21	0.3 (2)	C28—C23—C22—C21	−0.2 (3)
C19—N5—C20—C21	−179.46 (15)	C24—C23—C22—C21	−179.69 (16)
N5—C28—C23—C22	3.6 (2)	C7—C8—C9—C4	−0.4 (3)
C27—C28—C23—C22	−177.06 (16)	C3—C4—C9—C8	178.97 (18)
N5—C28—C23—C24	−176.92 (15)	C5—C4—C9—C8	0.0 (3)
C27—C28—C23—C24	2.4 (2)	C15—C14—C13—C18	−0.5 (3)
C6—C7—C8—C9	0.1 (3)	C15—C14—C13—Cl2	179.42 (15)
C10—C7—C8—C9	−179.46 (17)	C17—C18—C13—C14	1.3 (3)
N5—C20—C21—C22	3.1 (3)	Cl1—C18—C13—C14	−179.92 (15)
N5—C20—C21—C29	−176.72 (16)	C17—C18—C13—Cl2	−178.67 (15)
C6—C7—C10—C12	177.58 (16)	Cl1—C18—C13—Cl2	0.1 (2)
C8—C7—C10—C12	−2.9 (3)	C5—C4—C3—C1	2.3 (3)
C6—C7—C10—C11	−3.8 (3)	C9—C4—C3—C1	−176.66 (18)
C8—C7—C10—C11	175.72 (17)	C5—C4—C3—C2	178.20 (18)
C4—C5—C6—C7	−1.3 (3)	C9—C4—C3—C2	−0.7 (3)
C10—C7—C6—C5	−179.66 (16)	C22—C23—C24—C25	177.83 (18)
C8—C7—C6—C5	0.8 (3)	C28—C23—C24—C25	−1.6 (3)
C18—C17—C16—C15	−0.4 (3)	C13—C14—C15—C16	−0.7 (3)
C18—C17—C16—C19	−178.37 (16)	C17—C16—C15—C14	1.1 (3)
N5—C28—C27—C26	177.88 (16)	C19—C16—C15—C14	179.13 (17)
C23—C28—C27—C26	−1.4 (3)	C23—C24—C25—C26	−0.1 (3)
C20—N5—C19—C16	−100.94 (17)	C28—C27—C26—C25	−0.4 (3)
C28—N5—C19—C16	79.31 (18)	C24—C25—C26—C27	1.2 (3)

### Hydrogen-bond geometry (Å, °)

**Table:**

*D*—H···*A*	*D*—H	H···*A*	*D*···*A*	*D*—H···*A*
C20—H20···N3^i^	0.93	2.53	3.387 (3)	154
C19—H19B···N3^i^	0.97	2.51	3.432 (2)	158
C14—H14···N3^ii^	0.93	2.50	3.390 (3)	161
C15—H15···N1^iii^	0.93	2.45	3.348 (2)	163

Symmetry codes: (i) *x*, *y*, *z*−1; (ii) *x*−1, *y*, *z*−1; (iii) −*x*, −*y*+1, −*z*+1.
